# Supplementary material for: Identifying the Barriers to Universal Cervical Length Screening for Preterm Birth Prevention at a Tertiary Hospital in Thailand (Physician Perspectives): Implementation Research
Source: Healthcare (Basel). 2023 Apr 4;11(7):1039. doi: 10.3390/healthcare11071039 (PMC10093914; doi:10.3390/healthcare11071039)
Supplement: Supplementary file 1 [file healthcare-11-01039-s001.zip › healthcare-2196431 TablesS1-S7.pdf]

Table S1. Context evaluation of tertiary hospitals (more than one option able to be selected)

Relevant content	Number (%) (n = 120)	95% Confidence interval
Concrete hospital policy for cervical length screening to prevent preterm births:		
• There is a specific operating policy	• 55 (45.8%)	• 37.2% to 54.7%
• Screening is done in parallel with other duties (e.g., teaching; research; etc.)	• 32 (26.7%)	• 19.6% to 35.2%
• No policy	• 33 (27.5%)	• 20.3% to 36.1%
Working group or committee in place to implement preterm birth prevention:		
• Working group established	• 31 (25.8%)	• 18.8% to 34.3%
• Only some personnel are assigned	• 52 (43.3%)	• 34.8% to 52.3%
• No assignment	• 37 (30.9%)	• 23.3% to 39.6%
Action plan to prevent preterm births in the hospital:		
• Yes	• 108 (90.0%)	• 83.3% to 94.2%
• No	• 12 (10.0%)	• 5.8% to 16.7%
If action plan is established (n = 108/120):		
• There is a monthly/quarterly/yearly operational planning meeting	• 54/108 (50.0%)	• 40.7% to 59.3%
• There is a meeting to report performance monthly/quarter/yearly	• 58/108 (53.7%)	• 44.3% to 62.8%
• A meeting or an activity is held occasionally	• 27/108 (25.0%)	• 17.8% to 33.9%
Have a role as a working physician in formulating policies relating to preterm birth prevention:		
• Yes	• 99 (82.5%)	• 74.7% to 88.3%
• No	• 21 (17.5%)	• 11.7% to 25.3%
Encouragement is given by the Maternal and Child Health Board for the conduct of a cervical length screening program at the hospital:		
• No	• 42 (35.0%)	• 27.1% to 43.9%
• Yes	• 78 (65.0%)	• 56.1% to 73.0%
Support is provided by the Maternal and Child Health Board for the implementation of a program of preterm birth prevention (n = 78/120):		
• Micronized Progesterone vaginal soft-gel capsules (Utrogestan)	• 46/78 (59.0%)	• 47.9% to 69.2%
• Progesterone pessaries (Cyclogest)	• 0 (0%)	• 0.0% to 4.7%
• 17-OHPC (Proluton Depot)	• 37/78 (47.4%)	• 36.7% to 58.4%
• Funding for training of medical personnel in cervical length measurement	• 19/78 (24.4%)	• 16.2% to 34.9%
• Funding for the purchase of ultrasound equipment	• 14/78 (17.9%)	• 11.0% to 27.9%
• Other	• 2/78 (2.6%)	• 0.7% to 8.9%
Perception of the current role of the Maternal and Child Health Board in a cervical length screening program to prevent preterm births:		
• No role at all	• 24 (20.0%)	• 13.8% to 28.0%
• Limited role	• 59 (49.2%)	• 40.4% to 58.0%
• Very active	• 37 (30.8%)	• 23.3% to 39.6%
Roles of the Maternal and Child Health Board that you would like to see or receive:		
• Act as a policy maker and provide the main operation plans so that all hospitals operate in the same way and their performances can be compared	• 32 (80.0%)	• 65.2% to 89.5%
• Be the leader or individual responsible for an academic training program of cervical length screening for staff at a district hospital	• 24 (20.0%)	• 13.8% to 28.0%
• Responsible for providing medicines and medical supplies to all hospitals	• 59 (49.2%)	• 40.4% to 58.0%
• Other	• 37 (30.8%)	• 23.3% to 39.6%

Table S2. Availability of resources

Resources	Number (%) (n = 120)	95% Confidence interval
Hospital regularly employs an adequate number of obstetricians to meet workloads:<br>• No<br>• Yes	• 44 (36.7%)<br>• 76 (63.3%)	• 28.6% to 45.6%<br>• 54.4% to 71.4%
Hospital has obstetricians who can accurately perform cervical length measurements:<br>• No<br>• Yes, but not enough<br>• Yes, enough	• 3 (2.5%)<br>• 57 (47.5%)<br>• 60 (50.0%)	• 0.9% to 7.1%<br>• 38.8% to 56.4%<br>• 41.2% to 58.8%
Hospital has a person responsible for providing information on a preterm birth prevention program (Project Manager):<br>• Yes<br>• No	• 62 (51.7%)<br>• 58 (48.3%)	• 42.8% to 60.4%<br>• 39.6% to 57.2%
Hospital has a specific budget for cervical length measurement screening:<br>• Yes (funds are sourced from the district budget)<br>• No	• 13 (10.8%)<br>• 107 (89.2%)	• 6.4% to 17.7%<br>• 82.3% to 93.6%
Hospital has enough ultrasound machines that can be used for routine tasks:<br>• Not enough<br>• Enough	• 44 (36.7%)<br>• 76 (63.3%)	• 28.6% to 45.6%<br>• 54.4% to 71.4%
Hospital has an ultrasound machine that can be used specifically for a cervical length measurement screening program:<br>• No<br>• Yes	• 59 (49.2%)<br>• 61 (50.8%)	• 40.4% to 58.0%<br>• 42.0% to 59.6%

Table S3. Impact of preterm births on hospitals, pregnant women, and families

Hospital aspect	Number (%) (n = 120)	95% Confidence interval
Workload of the personnel involved:<br>• No effect<br>• Little effect<br>• Heavy effect	• 2 (1.7%)<br>• 22 (18.3%)<br>• 96 (80.0%)	• 0.5% to 5.9%<br>• 12.4% to 26.2%<br>• 72.0% to 86.2%
Expenses that the hospital has to bear:<br>• No effect<br>• Little effect<br>• Heavy effect	• 3 (2.5%)<br>• 16 (13.3%)<br>• 101 (84.2%)	• 0.9% to 7.1%<br>• 8.4% to 20.6%<br>• 76.6% to 89.6%
Length of hospital stays:<br>• No effect<br>• Little effect<br>• Heavy effect	• 7 (5.8%)<br>• 20 (16.7%)<br>• 93 (77.5%)	• 2.9% to 11.6%<br>• 11.1% to 24.4%<br>• 69.2% to 84.1%
**Pregnant women and family aspects**		

Family expenses:<br>• No effect<br>• Little effect<br>• Heavy effect	 • 2 (1.7%)<br>• 22 (18.3%)<br>• 96 (80.0%)	 • 0.5% to 5.9%<br>• 12.4% to 26.2%<br>• 72.0% to 86.2%
Problems with caring for the newborn:<br>• No effect<br>• Little effect<br>• Heavy effect	 • 1 (0.8%)<br>• 8 (6.7%)<br>• 111 (92.5%)	 • 0.2% to 4.6%<br>• 3.4% to 12.6%<br>• 86.4% to 96.0%
Problems in the life and work of the family members:<br>• No effect<br>• Little effect<br>• Heavy effect	 • 2 (1.7%)<br>• 11 (9.2%)<br>• 107 (89.2%)	 • 0.5% to 5.9%<br>• 5.2% to 15.7%<br>• 82.3% to 93.6%

Table S4. Assessment of project inputs

Input evaluation	Number (%) (n = 120)	95% Confidence interval
Realization at the hospital that preterm births present problems:<br>• Yes<br>• No	 • 119 (99.2%)<br>• 1 (0.8%)	 • 95.4% to 99.9%<br>• 0.2% to 4.6%
Overall degree of severity of the problems:<br>• Low<br>• Moderate<br>• High	 • 4 (3.4%)<br>• 52 (43.7%)<br>• 63 (52.9%)	 • 1.3% to 8.3%<br>• 34.8% to 52.3%<br>• 43.6% to 61.2%
Physicians can perform cervical length measurements by vaginal ultrasound:<br>• Yes (with related certification)<br>• Yes (without related certification)<br>• No	 • 19 (15.8%)<br>• 96 (80.0%)<br>• 5 (4.2%)	 • 10.4% to 23.4%<br>• 72.0% to 86.2%<br>• 1.8% to 9.4%
Usefulness of cervical length measurements to prevent preterm births (physicians' perspectives):<br>• Useless<br>• Somewhat useful<br>• Very useful	 • 0 (0%)<br>• 30 (25.0%)<br>• 90 (75.0%)	 • 0.0% to 3.1%<br>• 18.1% to 33.4%<br>• 66.6% to 81.9%
If cervical length measurements to prevent preterm births are deemed somewhat useful or useless (physicians' perspectives; n = 30/120):<br>• Perform cervical length measurements only in high-risk cases<br>• Use other screening tests; advise bed rest and drug usage<br>• Other	 • 8/30 (26.6%)<br>• 3/30 (10.0%)<br>• 19/30 (63.4%)	 • 14.2% to 44.5%<br>• 3.5% to 25.6%<br>• 45.5% to 78.1%
If cervical length measurements to prevent preterm births are deemed very useful (physicians' perspectives; n = 90/120):<br>• Establish a hospital policy to do as a stand-alone task<br>• Establish a hospital policy to do in conjunction with other screening tasks<br>• A hospital policy not required; screening can be performed as needed	 • 40/90 (44.4%)<br>• 46/90 (51.1%)<br>• 5/90 (5.6%)	 • 34.6% to 54.7%<br>• 41.0% to 61.2%<br>• 2.4% to 12.4%

Universal cervical length screening helps to reduce preterm births (n = 116/120):<br>• Agree; it would reduce the rate of preterm births<br>• Disagree; it would not affect the rate of preterm births	• 76/116 (65.5%)<br>• 40/116 (34.5%)	• 56.5% to 73.5%<br>• 26.5% to 43.5%
Awareness of the policy for universal cervical measurements for premature birth prevention included in the 2017 policy guidelines of the Ministry of Public Health:<br>• Aware<br>• Was not aware	• 88 (73.3%)<br>• 32 (26.7%)	• 64.8% to 80.4%<br>• 19.6% to 35.2%
If aware of the policy, level of agreement with the policy (n = 88/120):<br>• Agree<br>• Disagree<br>• Other	• 55/88 (62.5%)<br>• 20/88 (22.7%)<br>• 13/88 (14.8%)	• 52.1% to 71.9%<br>• 15.2% to 32.5%<br>• 8.8% to 23.7%
A fee for ultrasound screening:<br>• Should not be charged to pregnant women<br>• Should be charged to pregnant women	• 91 (75.8%)<br>• 29 (24.2%)	• 67.5% to 82.6%<br>• 17.4% to 32.6%
Willingness to work on a project to promote full implementation of universal cervical length screening:<br>• Not willing<br>• Willing	• 26 (21.7%)<br>• 94 (78.3%)	• 15.2% to 29.9%<br>• 70.2% to 84.8%
If not willing to work on the project (n = 26/120):<br>• Already have too much, or enough, full-time work<br>• Already have a substantial workload other than the regular job<br>• Do not think the preterm birth rate is such a severe problem that a program is needed<br>• Do not believe that cervical length screening plays a role in preventing preterm births<br>• Do not believe that cervical length screening to prevent preterm births is worth the requisite labor or funding<br>• Unsure of the accuracy of cervical length measurements<br>• Other	• 20/26 (76.9%)<br>• 14/26 (53.8%)<br>• 2/26 (7.7%)<br>• 3/26 (11.5%)<br>• 10/26 (38.5%)<br>• 7/26 (26.9%)<br>• 4/26 (15.4%)	• 58.0% to 89.0%<br>• 35.5% to 71.2%<br>• 2.1% to 24.1%<br>• 4.0% to 29.0%<br>• 22.4% to 57.5%<br>• 13.7% to 46.1%<br>• 6.2% to 33.5%

Table S5. Process evaluation of universal cervical length screening program

Process evaluation	Number (%) (n = 120)	95% Confidence interval
With reference to the policy of the Ministry of Public Health for universal cervical length screening to prevent preterm births, the situation in the hospital is:<br>• Screening is performed for every case<br>• Screening is performed for high-risk cases only<br>• No screening tests are performed	• 29 (24.2%)<br>• 73 (60.8%)<br>• 18 (15.0%)	• 17.4% to 32.6%<br>• 51.9% to 69.1%<br>• 9.7% to 22.5%
With reference to hospital guidelines for the performance of cervical length screening to prevent preterm births:<br>• Hospital guidelines are systematic and clear<br>• The content of hospital guidelines is inadequate or unclear (e.g., because of an over-reliance on an unhelpful format, such as a flow chart)<br>• No hospital guidelines have been established	• 33 (27.5%)<br>• 73 (60.8%)<br>• 14 (11.7%)	• 20.3% to 36.1%<br>• 51.9% to 69.1%<br>• 7.1% to 18.6%
If systematic guidelines or documentation is provided by the hospital, the situation with the real practice of following those guidelines and documents is (n = 106):<br>• They are not observed<br>• Only some parts are observed, or they are observed in whole only occasionally<br>• They are strictly observed	(n = 106)<br>• 1/106 (0.9%)<br>• 79/106 (74.5%)<br>• 26/106 (24.5%)	• 0.2% to 5.2%<br>• 65.5% to 81.9%<br>• 17.3% to 33.5%

If systematic guidelines or documentation is provided by the hospital, specific workloads are set for each physician (n = 106):<br>• Yes<br>• No	(n = 106)<br>• 49/106 (46.2%)<br>• 57/106 (53.8%)	 • 37.0% to 55.7%<br>• 44.3% to 63.0%
With reference to the broad policies of the Ministry of Public Health relating to the prevention of preterm births, the hospital has implemented those policies:<br>• Yes, it has<br>• No, it has not	• 89 (74.2%)<br>• 31 (25.8%)	• 65.7% to 81.2%<br>• 18.8% to 34.3%
There are problems when screening is performed for the target group (pregnant women who are at risk):<br>• No<br>• Yes<br>Problems encountered:<br>▪ Long waiting times<br>▪ Unsatisfactory service<br>▪ Cost<br>▪ Shyness/fear of examination/fear of pain<br>▪ Other (e.g., the patient declined a transvaginal ultrasound; late antenatal care; unable to attend appointment; delays associated with patient-preparation)	• 46 (38.3%)<br>• 74 (61.7%)<br><br>▪ 54 (45.0%)<br>▪ 12 (10.0%)<br>▪ 24 (20.0%)<br>▪ 40 (33.3%)<br>▪ 14 (11.7%)	• 30.1% to 47.3%<br>• 52.7% to 69.9%<br><br>▪ 36.4% to 53.9%<br>▪ 5.8% to 16.7%<br>▪ 13.8% to 28.0%<br>▪ 25.5% to 42.2%<br>▪ 7.1% to 18.6%

Table S6. Possible barriers to universal cervical length screening at hospitals

Possible barriers	Number (%) (n = 120)	95% Confidence interval
Problems related to the physicians:<br>• Insufficient number of doctors available to perform the procedure<br>• Doctors have other urgent and necessary tasks<br>• Doctors have excessive routine tasks<br>• Doctors do not think that premature births are such a severe problem that the scheme is required<br>• Doctors do not think that cervical length screening plays a role in preventing preterm births<br>• Doctors do not believe that universal cervical length screening to prevent preterm births can justify the requisite labor and funding<br>• Doctors are unsure about the correct procedures for the measurements<br>• Other	 • 51 (42.5%)<br>• 63 (52.5%)<br>• 78 (65.0%)<br>• 4 (3.3%)<br>• 28 (23.3%)<br>• 37 (30.8%)<br>• 50 (41.7%)<br>• 9 (7.5%)	 • 34.0% to 51.4%<br>• 43.6% to 61.2%<br>• 56.1% to 73.0%<br>• 1.3% to 8.3%<br>• 16.7% to 31.7%<br>• 23.3% to 39.6%<br>• 33.2% to 50.6%<br>• 4.0% to 13.6%
Problems related to other personnel, such as nurses and administrative staff:<br>• Insufficient number of personnel to support the performance of the procedure<br>• There are other tasks that are more urgent<br>• The staff already have an excessive volume of routine tasks to perform<br>• Lack of confidence that the collecting, recording, and analyzing of the data by non-medical personnel will be accurate<br>• Other	 • 75 (62.5%)<br>• 38 (31.7%)<br>• 72 (60.0%)<br>• 51 (42.5%)<br>• 2 (1.7%)	 • 53.6% to 70.7%<br>• 24.0% to 40.5%<br>• 51.1% to 68.3%<br>• 34.0% to 51.4%<br>• 0.5% to 5.9%

Problems related to the hospital:		
• Hospital administrators ignore the issue	• 21 (17.5%)	• 11.7% to 25.3%
• Lack of support for operating funds from government agencies	• 67 (55.8%)	• 46.9% to 64.4%
• Lack of cost-free drug support (progesterone) for pregnant women with short cervices to prevent preterm births	• 62 (51.7%)	• 42.8% to 60.4%
• Other	• 14 (11.7%)	• 7.1% to 18.6%

Table S7. Possible approaches to surmounting obstacles

Possible ways	Number (%) (n = 120)	95% Confidence interval
Relevant to physicians and/or related persons:		
• Add/request additional doctors who have the potential to screen cervical lengths using various methods	• 42 (35.0%)	• 27.1% to 43.9%
• Provide regular training to physicians to enable them to confidently measure cervical lengths	• 68 (56.7%)	• 47.7% to 65.2%
• Provide knowledge and skills relating to cervical length measurements for doctors who perform routine work so that they can become certificated and undertake examinations confidently	• 68 (56.7%)	• 47.7% to 65.2%
• Provide reliable research results/demonstrations of the procedure/examples of screening results, and present doctors/nurses/other stakeholders with a detailed and convincing case for the cost-effectiveness of implementing universal cervical length screening	• 48 (40.0%)<br><br>• 51 (42.5%)	• 31.7% to 48.9%<br><br>• 34.0% to 51.4%
• Reduce extraneous duties for doctors	• 5 (4.2%)	• 1.8% to 9.4%
• Other		
Relevant to hospitals:		
• Provide hospitals with adequate and regular funding from relevant agencies	• 92 (76.7%)	• 68.3% to 83.3%
• Extend screening to community hospitals to relieve workloads at tertiary centers	• 84 (70.0%)	• 61.3% to 77.5%
• Educate patients about the benefits of cervical length measurements to prevent preterm births	• 78 (65.0%)	• 56.1% to 73.0%
• Other (providing adequate ultrasound machines and, in high-risk cases, administering appropriate preventative medications)	• 4 (3.3%)	• 1.3% to 8.3%
Monitoring of the systems related to, and evaluation of the results of, providing universal cervical length screening by the abovementioned approaches:		
• No aspects identified	• 56 (46.7%)	• 38.0% to 55.6%
• Some aspects identified (n = 53)	• 53 (44.2%)	• 35.6% to 53.1%
▪ Objective	▪ 27/53 (50.9%)	▪ 37.9% to 63.9%
▪ Monitoring system	▪ 9/53 (17.0%)	▪ 9.2% to 29.2%
▪ Evaluation of screening results	▪ 16/53 (30.2%)	▪ 19.5% to 43.5%
▪ Treatment evaluation	▪ 15/53 (28.3%)	▪ 18.0% to 41.6%
▪ Assessment of the incidence of preterm births after project completion	▪ 15/53 (28.3%)	▪ 18.0% to 41.6%
• All aspects	• 11 (9.2%)	• 5.2% to 15.7%
