# Supplementary material for: Identifying the Barriers to Universal Cervical Length Screening for Preterm Birth Prevention at a Tertiary Hospital in Thailand (Physician Perspectives): Implementation Research
Source: Healthcare (Basel). 2023 Apr 4;11(7):1039. doi: 10.3390/healthcare11071039 (PMC10093914; doi:10.3390/healthcare11071039)
Supplement: Supplementary file 1 [file healthcare-11-01039-s001.zip › healthcare-2196431-File S1.pdf]

Participant number.....................

Questionnaire for Practitioner Obstetricians

Research project: identifying barriers to universal cervical length screening for the prevention of preterm births: implementation research

Overview

This questionnaire is divided into 4 parts

Part 1 Personal Information

Part 2 Context Evaluation

Part 3 Input Evaluation

Part 4 Process Evaluation

Part 1 Personal Information

1. Your current age .... years
2. Obtained MD .... years ago
3. Completed OB&GYN board 1 Yes 0 No
4. Completed MFM board 1 Yes 0 No
5. Have worked in obstetrics and gynecology for .... years
6. Level of hospital

    1 30–60 .... beds

    2 60–120 .... beds

    3 120–300 .... beds

    4 300–500 .... beds

    5 > 500 .... beds

7. Duties other than working as a doctor. Please tick ✓ the box ☐ in front of the relevant item.

    ☐ Administrative work (other than as the department or unit head)

    ☐ Teaching work

☐ Research work

☐ Other (specify) ....

Part 2 Context Evaluation

1. Does your hospital currently have a policy to measure the length of the cervix for preterm birth prevention?

   ☐ Yes, there is a specific operating policy

   ☐ There is a policy to operate in conjunction with other tasks

   ☐ No policy

   Additional comments ....

2. Does your hospital have a working group/committee responsible for the prevention of preterm births?

   1 A clear working group/committee has been established.

   2 A working group is formed from time to time on an ad hoc basis to consider the prevention of preterm births

   3 No working group/committee has been established.

   4 Other (specify) ....

3. Does your hospital have an action plan to prevent preterm births?

   1 Yes 2 No

   If yes, please tick ✓ in the box ☐ in front of the relevant item

   ☐ Monthly/Quarterly/Annual Planning Meeting

   ☐ Performance report meeting Monthly/Quarterly/Yearly

   ☐ Meeting or activity

   ☐ Other (specify) ....

   If there is a prepared action plan, please tick ✓ the box ☐ in front of the relevant item(s). You can choose more than one item.

   ☐ Meetings to clarify the agency's guidelines for preventing premature births

☐ Short-term academic training for relevant personnel

☐ Sending doctors to train as residents and fellows in related fields

☐ Procurement of materials, equipment, and tools used in screening

☐ Procurement of drugs and medical supplies

☐ Other (specify) ....

4. As a medical practitioner, do you have a role to play in formulating preterm birth prevention policies?

   1 Yes 2 No

   Other ideas ....

5. Do you think that the Maternal and Child Health Board member in the region where your hospital is located provides any support for the cervical length screening program in your hospital?

   1 No support is provided (move on to question 7)

   2 Support is provided (continue to question 6)

6. In what areas have you received support from the Maternal and Child Health Board to use in preterm birth prevention policy? You can choose more than one item. Please tick ✓ the box ☐ in front of the relevant item(s).

   ☐ Free medicines are provided, namely:

      ☐ Micronized Progesterone vaginal soft-gel capsules (Utrogestan)

      ☐ Progesterone pessaries (Cyclogest)

      ☐ 17-OHPC (Proluton depot)

   ☐ Budget for medical personnel training in cervical length measurement training

   ☐ Budget for the purchase of an ultrasound machine to measure the length of the cervix

   ☐ Other (specify) ....

7. In practice, how substantial is the role of the Maternal and Child Health Board partner in your area in your hospital's cervical length screening program to prevent preterm birth?

   1 No role at all

2 Little Role

3 Plays a big role

If there is a role, please specify..

8. What role would you like the Maternal and Child Health Board to have in preterm birth prevention?

    8.1 To ensure that the policies and the main operating plans of all hospitals in the district are the same, and to compare their performance.

    8.2 To be a leader/responsible for providing academic training to hospital personnel in the district

    8.3 To be responsible for supplying medicines, materials, medical equipment to all hospitals in the district.

9. Availability of resources

    9.1 Do you think your hospital has enough obstetricians to perform their full-time jobs?

    1 Insufficient 2 Sufficient

    9.2 Does your hospital have an obstetrician who can measure the length of the cervix accurately enough?

    1 No 2 Insufficient 3 Sufficient

    9.3 Does your hospital have a specific person responsible for the preterm birth prevention program (a project manager)?

    1 Yes

    2 No-one is responsible

    9.4 Does your hospital have a specific budget for cervical length screening?

    1 Yes; the budget comes from (please specify) ....

    2. There is no budget.

    9.5 Does your hospital have enough ultrasound machines that can be used for routine tasks?

    1 Insufficient

    2 Sufficient

9.6 Does your hospital have enough ultrasound machines that can be used specifically for a cervical length screening program?

1 Insufficient

2 Sufficient

10. To what extent do you think your hospital is affected by preterm births in the following area?

10.1 A greater workload for the personnel involved

1 No effect 2 Slight effect 3 A substantial impact

10.2 Expenses that the hospital has to bear

1 No effect 2 Slight effect 3 A substantial impact

10.3 Long hospital stays

1 No effect 2 Slight effect 3 A substantial impact

10.4 Other ideas (specify) ....

11. To what extent do you think the patient's family is affected by the following issues?

11.1 Expense

1 No effect 2 Slight effect 3 A substantial impact

11.2 Problems with newborn care

1 No effect 2 Slight effect 3 A substantial impact

11.3 Problems with daily life and work for the family members

1 No effect 2 Slight effect 3 A substantial impact

11.4 Other comments (specify) ....

Part 3 Input Evaluation

1. Do you think premature births present problems to your hospital?

1 Yes 2 No

1.1 If yes, what is the severity of the problems?

1 Little 2 Moderate 3 A substantial impact

2. Can you measure the length of the cervix with a vaginal ultrasound machine?

2.1 Can do it, and I have a certificate of competence

2.2 Can do it, but I do not have a certificate of competence

2.3 Cannot do it

3. Do you find it helpful to measure the length of the cervix to prevent preterm births?

1 Not helpful 2 Moderately helpful 3 Very helpful

3.1 If you think it is not helpful or only moderately helpful, what better method should be used instead to prevent premature births? (Specify)

....

3.2 If you think performing cervical length measurements is very helpful, which of the following do you think should be undertaken?

3.2.1 Establish a policy for the organization whereby cervical length measurements are routinely performed

3.2.2 Establish a policy for the organization whereby screening is performed in conjunction with other tasks.

3.2.3 Routine screening does not need to be defined as the organization's policy, but screening can be conducted as required by a doctor

4. Do you think universal screening for cervical length will reduce your hospital's preterm birth rate?

1 Yes

2 No

Other comments ....

5. Do you know the policy guidelines for universal screening of cervical length to prevent preterm birth contained in the guidelines of the Ministry of Public Health for the year 2017?

1 1 Yes 2 No

5.1 If known, do you agree with this policy?

1Agree because (specify) ....

2Disagree because (specify) ....

3Other (specify) ....

6. Do you think that universal cervical length screening should be waived for all pregnant women?

2 Yes 2 No

7. If your hospital offered a cervical length measurement program for the prevention of preterm births, would you be willing to participate in the program?

    1 Willing to participate

    2 Not willing to participate

    7.1 In the event that you are not willing to participate, why is that so? You can choose more than one item.

        7.1.1 Already have more than enough work in your full-time job

        7.1.2 There is already a lot of regular work.

        7.1.3 I don't think that the hospital's preterm birth rate is a serious enough problem to warrant the need for a program.

        7.1.4 I don't think that measuring the length of the cervix actually helps to prevent preterm births.

        7.1.5 I don't think that measuring the length of the cervix is worth the requisite investment in labor and funding for the prevention of preterm births.

        7.1.6 I am not confident in the accuracy of cervical length measurements made by me

        7.1.7 Other (specify)

        ....

Part 4. Process evaluation

1. Does your hospital have cervical length screening to prevent preterm births in accordance with the policy of the Ministry of Health?
2. Is the screening provided for all patients or only for high-risk patients?

    1. The screening test is provided for everyone.

    2. The screening test is performed only for high-risk patients.

    3 There is no screening test

    4 Additional comments ....

3. Does your hospital have practice guidelines for cervical length screening to prevent preterm births?

    1 Practice guidelines are organized in a systematic and clear manner.

    2Practice guidelines are presented in a non-systematic format, for example, in the form of a condensed document, flow chart, or the like.

    3 No practice guidelines have been prepared.

    4 More information..

    3.1 In the event that the practice guidelines are prepared in various forms, is work actually performed as specified?

        1 The practice guidelines are not observed at all.

        2 The practice guidelines are either partly observed or are only occasionally observed.

        3 The practice guidelines are strictly followed.

    3.2 In case of screening the length of the cervix, has your hospital clearly defined the duties of each operator?

        1 The duties have been satisfactorily defined

        2 The duties have not been satisfactorily defined

4. Does your hospital perform cervical length measurements to prevent premature births by using guidelines consistent with the policy of the Ministry of Public Health for the year 2017?

    1 Yes, it does.

    2 No, it doesn't.

    Additional comments..

5. Have you ever encountered problems in providing services to the target group (pregnant women who are at risk)?

    1 No

    2 Yes (please specify)...................

5.1 Long waiting time	1 Yes 2 No
5.2 Unsatisfactory service	1 Yes 2 No
5.3 Cost	1 Yes 2 No
5.4 Shyness/fear of examination/fear of pain	1 Yes 2 No

5.5 Other (specify) ....

6. What is the major obstacle to universal cervical length screening at your hospital? You can choose more than one item.

6.1 Problems related to the practitioners

6.1.1 There are not enough doctors to enable screening to be conducted.

6.1.2 Doctors have other tasks that are more urgent and necessary.

6.1.3 Doctors already have excessive workloads.

6.1.4 Doctors don't think preterm births are a serious enough problem to warrant this program.

6.1.5 Doctors do not think that measuring the length of the cervix actually plays a role in preventing preterm births.

6.1.6 Doctors do not think that measuring the length of the cervix to prevent preterm births is a cost-effective investment of labor or funds.

6.1.7 Doctors are not confident in the accuracy of cervical length measurements they or other doctors have made.

6.1.8 Other (specify) ....

6.2 Problems related to other personnel such as nurses, staff

6.2.1 There are not enough personnel to enable screening to be conducted.

6.2.2 There are other tasks that are more urgent and necessary.

6.2.3 Excessively sedentary

6.2.4 Unreliable accuracy in collecting, recording, and analyzing information by non-medical personnel

6.2.5 Other (specify) ....

6.3 Problems related to the hospital

6.3.1 The hospital administrators do not pay attention to this problem.

6.3.2 Lack of budgetary support from government agencies

6.3.3 Lack of support for free drugs to prevent preterm births by women with a short cervix

6.3.4 Other (specify)

....

6.4 Any other issues ....

7. Among the problems and obstacles above, which one item do you think represents the main obstacle to successful universal cervical screening at your hospital?

   ………

8. How do you think the above obstacles can be solved? You can choose more than one item.

   8.1 Regarding the doctors or related personnel

   8.1 .1 Add/request more potential physicians to conduct screening

   8.1.2 Full-time physicians must be trained in various methods of cervical length measurements

   8.1.3 Train physicians so that they can confidently screen the length of the cervix.

   8.1.4 Develop knowledge and skills in the techniques of cervical length measurement for practitioners until they are confident and are certified in an examination

   8.1.5 Provide doctors, nurses, and other stakeholders with reliable research results, operating results, and examples so that they become aware of the cost-effectiveness of the procedure.

   8.1.6 Reduce the burden of unnecessary patient care.

   8.1.7 Other solutions (specify)

   ……………………………………………………………………………………………………

   8.2 Regarding the hospitals

   8.2.1 Relevant agencies must provide hospitals with adequate and consistent budgetary support.

   8.2.2 Physicians should be fully trained so that they can provide prompt universal cervical length screening.

   8.2.3 Educate patients on the benefits of cervical length measurement to prevent preterm births

   8.2.4 Other solutions (specify) ……………………………………………………………………

9. If your hospital has a system for monitoring and evaluating the results of the universal cervical length screening,

   1 not all aspects listed below are available.

   2 only some aspects listed below are available (specify) …………………………

   3 all aspects listed below are available.

9.1 Targets

9.2 Monitoring system

9.3 Screening evaluation

9.4 Treatment evaluation

9.5 Assessment of the incidence of preterm birth after the program

9.6 Any other problem (specify)

....

10. Comments

....

....

....

....
